# An Efficient Method to Calculate Genomic Prediction Accuracy for New Individuals

**DOI:** 10.3389/fgene.2019.00596

**Published:** 2019-06-25

**Authors:** Mohammad H. Ferdosi, Natalie K. Connors, Bruce Tier

**Affiliations:** Animal Genetics and Breeding Unit, University of New England, Armidale, NSW, Australia

**Keywords:** accuracy, efficient, breeding value, prediction, method

## Abstract

Diagonal elements of the coefficient matrix are necessary to calculate the genomic prediction accuracy. Here an improved methodology is described, to update the inverse of the coefficient matrix (**C**) for new individuals with a genotype, with and without phenotypes. Computational performance is significantly improved by re-using parts of the coefficient matrix inverse calculations that do not change from one animal to another, in combination with updated calculations for those that do change. This method expedites calculation of accuracy for new individuals with genotypes, without re-doing the whole population, by using the previously calculated matrices.

## 1. Introduction

In the last decade, technological advances have significantly decreased genotyping costs, particularly for agricultural livestock and cropping species. This reduction in costs has enabled regular genomic best linear unbiased prediction (GBLUP) (VanRaden, 2008) analyses for the production of genomic breeding values. Currently, genomic information is routinely used in the Australian beef industry in producing estimated breeding values (EBVs). Low costs and high industry uptake has resulted in a rapid increase in the number of new genotypes and thus the size of the genomic population is growing larger. GBLUP requires inversion of the genomic relationship matrix (**G**) and the coefficient matrix (**C**), which is computationally demanding. More efficient methods such as APY (Algorithm for Proven and Young animals) (Misztal, 2016) and PICD (Partial Incomplete Cholesky Decomposition) (Hancock, 2017) can handle large numbers of genotyped animals by approximating the inverse of **G** only and not the coefficient matrix. However, these approaches do not address the need for diagonal elements of the coefficient matrix inverse (left hand side) required for calculating EBV accuracies. With the increasing speed at which new genotypes are provided, inversion of the coefficient matrix for accuracy calculation is increasingly computationally demanding and time consuming, requiring more efficient methods.

Here we propose a method to calculate the accuracy of new individuals, with and without phenotypes, by updating the coefficient matrix inverse (**C**^−1^) for new individuals only, without re-doing the whole population. Using this method, we significantly reduce time and computational demand by updating the accuracy of new individuals and reducing redundancy in the reference population.

## 2. Methods

### 2.1. Theory

We consider a simple animal model without fixed effects. This model is

(1) $$\begin{array}{l} y=Zu+e \end{array}$$

where **y**, **Z**, **u**, and **e** are vector of observations, design matrix, vector of solutions, and vector of random residual effects, respectively. The solutions and residual variances are $var\left(\text{u}\right)=\text{G}\sigma_{u}^{2}$ and $var\left(\text{e}\right)=\text{I}\sigma_{e}^{2}$. The mixed model equations (MME) for the above model are

(2) $$\begin{array}{l} \text{Cu}=[Z^{\prime }Z+\alpha G^{-1}]u=r, \end{array}$$

where **C** is the coefficient matrix and $\alpha =\frac{\sigma_{e}^{2}}{\sigma_{u}^{2}}$. Henderson derived a method by using the diagonal of **C**^−1^ and the diagonal of **G** to calculate the accuracy of each estimated breeding value (Henderson, 1975). Accordingly, the accuracy can be calculated with this formula $\sqrt{1-\alpha \frac{{\text{c}}^{\text{ii}}}{{\text{g}}_{\text{ii}}}}$, where c^ii^ is the diagonal element of C^−1^ for individual i, and g_ii_ is the diagonal element of **G** for individual i.

#### 2.1.1. Updating C^−1^ to Illustrate the Proposed Method

To calculate the accuracy of individuals with or without phenotypes, each individual can be added to **C**^−1^ separately. In this case, the partitioned matrix of MME (Equation 2) is

(3) $$\begin{array}{l} \left[\begin{array}{cc} C_{\text{pp}} & C_{\text{pq}}\\ {\text{C}}_{\text{pq}}^{\prime } & C_{\text{qq}} \end{array}\right]\left[\begin{array}{c} u_{\text{p}}\\ u_{\text{q}} \end{array}\right]=\left[\begin{array}{c} r_{\text{p}}\\ r_{\text{q}} \end{array}\right] \end{array}$$

where subscript p and q are core individuals forming the reference population and new individuals respectively. New individuals may or may not have phenotypes.

As demonstrated in Equation (2), **Z′Z** becomes $\left(\begin{array}{cc} {\left({\text{Z}}^{\prime }\text{Z}\right)}_{\text{pp}} & 0\\ 0 & {\left({\text{Z}}^{\prime }\text{Z}\right)}_{\text{qq}} \end{array}\right)$ where ${\left({\text{Z}}^{\prime }\text{Z}\right)}_{\text{pp}}$ is a diagonal matrix with dimension equal to the number of core animals in the population, and the ${\left({\text{Z}}^{\prime }\text{Z}\right)}_{\text{qq}}$ in the lower diagonal represents new animals. Since **G**^−1^ becomes ${\left(\begin{array}{cc} {\text{G}}_{\text{pp}} & {\text{G}}_{\text{pq}}\\ {\text{G}}_{\text{pq}}^{\prime } & {\text{G}}_{\text{qq}} \end{array}\right)}^{-1}$, and **C**^−1^ becomes

(4) $$\begin{array}{l} {}[Z^{\prime }Z+\alpha G^{-1}{]}^{-1}\approx \\ \;{\left(\left[\begin{array}{cc} {\left(Z^{\prime }Z\right)}_{\text{pp}} & 0\\ 0 & {\left(Z^{\prime }Z\right)}_{\text{qq}} \end{array}\right]+\alpha {\left[\begin{array}{cc} {\text{G}}_{\text{pp}} & {\text{G}}_{\text{pq}}\\ {\text{G}}_{\text{pq}}^{\prime } & {\text{G}}_{\text{qq}} \end{array}\right]}^{-1}\right)}^{-1} \end{array}$$

based on Equations (2) and (3). Inverting **C** is computationally demanding, as both **G** and the entire **C** should be inverted in each analysis for all individuals. ${\text{G}}_{\text{pp}}^{-1}$ needs to be updated as the new individuals are added to **G**. This can be performed by the method explained in Meyer et al. (2013). However, since we want to know the accuracy, we must invert **C** as well as **G**. Equation (4) can be converted with the following inversion lemma which is equivalent to the Woodbury's formula (Henderson, 1963, 1975; Henderson and Searle, 1981):

(5) $$\begin{array}{l} {\left(A-BD^{-1}E\right)}^{-1}=E^{-1}D{\left(D-EA^{-1}B\right)}^{-1}EA^{-1}. \end{array}$$

With **C** we can consider ***A*** = **Z′Z**, **B** = −**I**, ***D***^−1^ = **G**^−1^ and **E** = α**I**. Thus **C**^−1^ is

(6) $$\begin{array}{l} {\left(Z^{\prime }Z+\alpha G^{-1}\right)}^{-1}=\alpha^{-1}G{\left(G+\alpha {\left(Z^{\prime }Z\right)}^{-1}\right)}^{-1}\alpha {\left(Z^{\prime }Z\right)}^{-1}\\ \;=GM^{-1}{\left(Z^{\prime }Z\right)}^{-1} \end{array}$$

where **M**^−1^ as (**G** + α(**Z′Z**)^−1^)^−1^ is used for simplification and is shown below in Equation (8). For the partitioned matrices in Equation (4), **C**^−1^ becomes

(7) $$\begin{array}{l} {\left(\left[\begin{array}{cc} {\left(Z^{\prime }Z\right)}_{\text{pp}} & 0\\ 0 & {\left(Z^{\prime }Z\right)}_{\text{qq}} \end{array}\right]+\alpha {\left[\begin{array}{cc} G_{\text{pp}} & G_{\text{pq}}\\ {\text{G}}_{\text{pq}}^{\prime } & G_{\text{qq}} \end{array}\right]}^{-1}\right)}^{-1}\\ \approx \left[\begin{array}{cc} G_{\text{pp}} & G_{\text{pq}}\\ {\text{G}}_{\text{pq}}^{\prime } & G_{\text{qq}} \end{array}\right]{\left(\left[\begin{array}{cc} G_{\text{pp}} & G_{\text{pq}}\\ {\text{G}}_{\text{pq}}^{\prime } & G_{\text{qq}} \end{array}\right]+\alpha \left[\begin{array}{cc} {\left(Z^{\prime }Z\right)}_{\text{pp}}^{-1} & 0\\ 0 & {\left(Z^{\prime }Z\right)}_{\text{qq}}^{-1} \end{array}\right]\right)}^{-1}\\ \;\left[\begin{array}{cc} {\left(Z^{\prime }Z\right)}_{\text{pp}}^{-1} & 0\\ 0 & {\left(Z^{\prime }Z\right)}_{\text{qq}}^{-1} \end{array}\right]\\ \approx \left[\begin{array}{cc} G_{\text{pp}} & G_{\text{pq}}\\ {\text{G}}_{\text{pq}}^{\prime } & G_{\text{qq}} \end{array}\right]{\left[\begin{array}{cc} G_{\text{pp}}+\alpha {\left(Z^{\prime }Z\right)}_{\text{pp}}^{-1} & G_{\text{pq}}\\ {\text{G}}_{\text{pq}}^{\prime } & G_{\text{qq}}+\alpha {\left(Z^{\prime }Z\right)}_{\text{qq}}^{-1} \end{array}\right]}^{-1}\\ \;\left[\begin{array}{cc} {\left(Z^{\prime }Z\right)}_{\text{pp}}^{-1} & 0\\ 0 & {\left(Z^{\prime }Z\right)}_{\text{qq}}^{-1} \end{array}\right], \end{array}$$

where ≈ is approximation sign. By using lemma (6) **G**^−1^ is not required and we only need to invert the middle matrix (**M**) in Equation (7). With this simplification **M**^−1^ can be updated for each new individual using Cholesky decomposition and multiplying the Cholesky factors, i.e., **M**^−1^ = **L**^−*T*^**L**^−1^ (Harville, 1997; Meyer et al., 2013).

(8) $$\begin{array}{l} M^{-1}=\left[\begin{array}{cc} L_{\text{pp}}^{-T}L_{\text{pp}}^{-1}+L_{\text{pp}}^{-T}{\text{L}}_{\text{qp}}^{\prime }L_{\text{qq}}^{-T}L_{\text{qq}}^{-1}L_{\text{qp}}L_{\text{pp}}^{-1} & -L_{\text{pp}}^{-T}{\text{L}}_{\text{pq}}^{\prime }L_{\text{qq}}^{-T}L_{\text{qq}}^{-1}\\ -L_{\text{qq}}^{-T}L_{\text{qq}}^{-1}L_{\text{qp}}L_{\text{pp}}^{-1} & L_{\text{qq}}^{-T}L_{\text{qq}}^{-1} \end{array}\right]\\ \;=[\begin{array}{cc} S_{1} & S_{2}\\ S_{3} & S_{4} \end{array}]. \end{array}$$

Therefore, Equation (7) can be written as

(9) $$\begin{array}{l} \;{\left[\begin{array}{cc} C_{\text{pp}} & C_{\text{pq}}\\ {\text{C}}_{\text{pq}}^{\prime } & C_{\text{qq}} \end{array}\right]}^{-1}=\left[\begin{array}{cc} G_{\text{pp}} & G_{\text{pq}}\\ {\text{G}}_{\text{pq}}^{\prime } & G_{\text{qq}} \end{array}\right]\;\left[\begin{array}{cc} S_{1} & S_{2}\\ S_{3} & S_{4} \end{array}\right]\;\left[\begin{array}{cc} {\left(Z^{\prime }Z\right)}_{\text{pp}}^{-1} & 0\\ 0 & {\left(Z^{\prime }Z\right)}_{\text{qq}}^{-1} \end{array}\right]\\ \;=\left[\begin{array}{cc} G_{\text{pp}}S_{1}+G_{\text{pq}}S_{3} & G_{\text{pp}}S_{2}+G_{\text{pq}}S_{4}\\ {\text{G}}_{\text{pq}}^{\prime }S_{1}+G_{\text{qq}}S_{3} & {\text{G}}_{\text{pq}}^{\prime }S_{2}+G_{\text{qq}}S_{4} \end{array}\right]\left[\begin{array}{cc} {\left(Z^{\prime }Z\right)}_{\text{pp}}^{-1} & 0\\ 0 & {\left(Z^{\prime }Z\right)}_{\text{qq}}^{-1} \end{array}\right]\\ =\left[\begin{array}{cc} (G_{\text{pp}}S_{1}+G_{\text{pq}}S_{3}){\left(Z^{\prime }Z\right)}_{\text{pp}}^{-1} & (G_{\text{pp}}S_{2}+G_{\text{pq}}S_{4}){\left(Z^{\prime }Z\right)}_{\text{qq}}^{-1}\\ ({\text{G}}_{\text{pq}}^{\prime }S_{1}+G_{\text{qq}}S_{3}){\left(Z^{\prime }Z\right)}_{\text{pp}}^{-1} & ({\text{G}}_{\text{pq}}^{\prime }S_{2}+G_{\text{qq}}S_{4}){\left(Z^{\prime }Z\right)}_{\text{qq}}^{-1} \end{array}\right], \end{array}$$

based on Equation (8) ${\text{S}}_{2}=-{\text{L}}_{\text{pp}}^{-T}{\text{L}}_{\text{qp}}^{\prime }{\text{L}}_{\text{qq}}^{-T}{\text{L}}_{\text{qq}}^{-1}$ and ${\text{S}}_{4}={\text{L}}_{\text{qq}}^{-T}{\text{L}}_{\text{qq}}^{-1}$. By multiplying back the Cholesky factors of **M** the solutions for ${\text{L}}_{\text{pq}}^{\prime }$ and ${\text{L}}_{\text{qq}}{\text{L}}_{\text{qq}}^{T}$ are

(10) $$\begin{array}{l} {\text{L}}_{\text{qp}}^{\prime }=L_{\text{pp}}^{-1}G_{\text{pq}} \end{array}$$

and

(11) $$\begin{array}{l} L_{\text{qq}}L_{\text{qq}}^{T}=G_{\text{qq}}+\alpha {\left(Z^{\prime }Z\right)}_{\text{qq}}^{-1}-{\text{G}}_{\text{pq}}^{\prime }{\left(G_{\text{pp}}+\alpha {\left(Z^{\prime }Z\right)}_{\text{pp}}^{-1}\right)}^{-1}G_{\text{pq}}, \end{array}$$

and **C**^qq^ which is the inverse of **C**_qq_ becomes

(12) $$\begin{array}{l} C^{\text{qq}}=({\text{G}}_{\text{pq}}^{\prime }(-L_{\text{pp}}^{-T}{\text{L}}_{\text{qp}}^{\prime }L_{\text{qq}}^{-T}L_{\text{qq}}^{-1})+G_{\text{qq}}(L_{\text{qq}}^{-T}L_{\text{qq}}^{-1})){\left(Z^{\prime }Z\right)}_{\text{qq}}^{-1}\\ \;=({\text{G}}_{\text{pq}}^{\prime }(-L_{\text{pp}}^{-T}{\text{L}}_{\text{pq}}^{\prime }S_{4})+G_{\text{qq}}S_{4}){\left(Z^{\prime }Z\right)}_{\text{qq}}^{-1}\\ \;=S_{4}({\text{G}}_{\text{pq}}^{\prime }(-L_{\text{pp}}^{-T}{\text{L}}_{\text{qp}}^{\prime })+G_{\text{qq}}){\left(Z^{\prime }Z\right)}_{\text{qq}}^{-1}\\ =\frac{-{\text{G}}_{\text{pq}}^{\prime }L_{\text{pp}}^{-T}{\text{L}}_{\text{qp}}^{\prime }+G_{\text{qq}}}{(G_{\text{qq}}+\alpha {\left(Z^{\prime }Z\right)}_{\text{qq}}^{-1}-{\text{G}}_{\text{pq}}^{\prime }{\left(G_{\text{pp}}+\alpha {\left(Z^{\prime }Z\right)}_{\text{pp}}^{-1}\right)}^{-1}G_{\text{pq}}){\left(Z^{\prime }Z\right)}_{\text{qq}}}\\ =\frac{-{\text{G}}_{\text{pq}}^{\prime }L_{\text{pp}}^{-T}L_{\text{pp}}^{-1}G_{\text{pq}}+G_{\text{qq}}}{(G_{\text{qq}}+\alpha {\left(Z^{\prime }Z\right)}_{\text{qq}}^{-1}-{\text{G}}_{\text{pq}}^{\prime }{\left(G_{\text{pp}}+\alpha {\left(Z^{\prime }Z\right)}_{\text{pp}}^{-1}\right)}^{-1}G_{\text{pq}}){\left(Z^{\prime }Z\right)}_{\text{qq}}} \end{array}$$

(13) $$\begin{array}{l} C^{\text{qq}}=\\ \frac{-{\text{G}}_{\text{pq}}^{\prime }L_{\text{pp}}^{-T}L_{\text{pp}}^{-1}G_{\text{pq}}+G_{\text{qq}}}{(G_{\text{qq}}+\alpha {\left(Z^{\prime }Z\right)}_{\text{qq}}^{-1}-{\text{G}}_{\text{pq}}^{\prime }{\left(G_{\text{pp}}+\alpha {\left(Z^{\prime }Z\right)}_{\text{pp}}^{-1}\right)}^{-1}G_{\text{pq}}){\left(Z^{\prime }Z\right)}_{\text{qq}}} \end{array}$$

where “—” is the right division sign (multiplying numerator by inverse of denominator) and ${\text{L}}_{\text{pp}}^{-T}{\text{L}}_{\text{pp}}^{-1}={\left({\text{G}}_{\text{pp}}+\alpha {\left({\text{Z}}^{\prime }\text{Z}\right)}_{\text{pp}}^{-1}\right)}^{-1}$ so

(14) $$\begin{array}{l} C^{\text{qq}}=\\ \frac{-{\text{G}}_{\text{pq}}^{\prime }{\left(G_{\text{pp}}+\alpha {\left(Z^{\prime }Z\right)}_{\text{pp}}^{-1}\right)}^{-1}G_{\text{pq}}+G_{\text{qq}}}{(G_{\text{qq}}+\alpha {\left(Z^{\prime }Z\right)}_{\text{qq}}^{-1}-{\text{G}}_{\text{pq}}^{\prime }{\left(G_{\text{pp}}+\alpha {\left(Z^{\prime }Z\right)}_{\text{pp}}^{-1}\right)}^{-1}G_{\text{pq}}){\left(Z^{\prime }Z\right)}_{\text{qq}}} \end{array}$$

Only ${\text{G}}_{\text{pq}}^{\prime }$, **G**_pq_, **G**_qq_, and ${\left({\text{Z}}^{\prime }\text{Z}\right)}_{\text{qq}}$ change with each new genotyped individual.

For animals without phenotypes ${\left({\text{Z}}^{\prime }\text{Z}\right)}_{\text{qq}}$ is a null matrix and the denominator in equation 14 becomes zero. However, we can assume the limit approach to α as ${\left({\text{Z}}^{\prime }\text{Z}\right)}_{\text{qq}}$ approach to zero. Thus, **C**^qq^ is

(15) $$\begin{array}{l} \underset{{\left(Z^{\prime }Z\right)}_{\text{qq}}\to 0}{\lim}\frac{-{\text{G}}_{\text{pq}}^{\prime }{\left(G_{\text{pp}}+\alpha {\left(Z^{\prime }Z\right)}_{\text{pp}}^{-1}\right)}^{-1}G_{\text{pq}}+G_{\text{qq}}}{(G_{\text{qq}}+\alpha {\left(Z^{\prime }Z\right)}_{\text{qq}}^{-1}-{\text{G}}_{\text{pq}}^{\prime }{\left(G_{\text{pp}}+\alpha {\left(Z^{\prime }Z\right)}_{\text{pp}}^{-1}\right)}^{-1}G_{\text{pq}}){\left(Z^{\prime }Z\right)}_{\text{qq}}}\\ \;=\frac{-{\text{G}}_{\text{pq}}^{\prime }{\left(G_{\text{pp}}+\alpha {\left(Z^{\prime }Z\right)}_{\text{pp}}^{-1}\right)}^{-1}G_{\text{pq}}+G_{\text{qq}}}{\alpha } \end{array}$$

In summary, Equations (14) and (15) can be used to calculate the prediction accuracies of individuals with and without phenotype, respectively.

#### 2.1.2. Updating the **M**^−1^ for New Individuals

Based on Equations (14) and (15) only ${\left({\text{G}}_{\text{pp}}+\alpha {\left({\text{Z}}^{\prime }\text{Z}\right)}_{\text{pp}}^{-1}\right)}^{-1}={\text{M}}_{\text{pp}}^{-1}$ changes (see Equation 7) in order to update the reference population. The updated ${\text{M}}_{\text{pp}}^{-1}$, i.e., ${\text{M}}_{{\text{pp}}_{new}}^{-1}$ is

(16) $$\begin{array}{l} M_{{\text{pp}}_{new}}^{-1}=\;\left[\begin{array}{cc} S_{n1} & S_{n2}\\ S_{n3} & S_{n4} \end{array}\right]\\ =\left[\begin{array}{cc} M^{-1}-S_{2}{\text{G}}_{\text{pq}}^{\prime }M^{-1} & -M^{-1}G_{\text{pq}}S_{4}\\ S_{n2}^{\prime } & {\left(G_{\text{qq}}+\alpha {\left(Z^{\prime }Z\right)}_{\text{qq}}^{-1}-{\text{G}}_{\text{pq}}^{\prime }M^{-1}G_{\text{pq}}\right)}^{-1} \end{array}\right] \end{array}$$

by regarding previous assumptions and Equation (8). **M**^−1^ is the largest matrix that was generated in the previous run and can be compressed and stored in binary format to avoid memory issues. The other matrices were small and can be built efficiently by using optimized Linear Algebra PACKage (LAPACK). The equations (14, 15, and 16) were implemented as an R function (Appendix A) to show the prototype and in C++ with Armadillo library (Sanderson and Curtin, 2016) to assess its performance—single thread.

#### 2.1.3. Simulated Data

Matrices with seven columns representing seven single-nucleotide polymorphisms for each individual and 1000, 2000, 3000, … 24000, and 25000 rows were created and filled with 0 (AA), 1 (AB), and 2 (BB) randomly. The genomic relationship matrices (**G**) were built by using VanRaden (2008) method 1, with dimension individuals by individuals. Importantly, increasing the number to SNPs does not affect the computational time. These matrices were used to assess the performance of the proposed method to calculate accuracy.

#### 2.1.4. Performance Evaluation

To evaluate performance, each set was run in three steps. In the first step, the elapsed time to build the coefficient matrix by using the classic approach (i.e., inverting **G** and **C**) was measured. In the second step, the time to build ${\left({\text{G}}_{\text{pp}}+\alpha {\left({\text{Z}}^{\prime }\text{Z}\right)}_{\text{pp}}^{-1}\right)}^{-1}$— initial matrices required to update c^qq^ was measured. In the third step, the time to calculate c^qq^ by using the initial matrices was measured.

## 3. Results and Discussions

**Figure 1:**
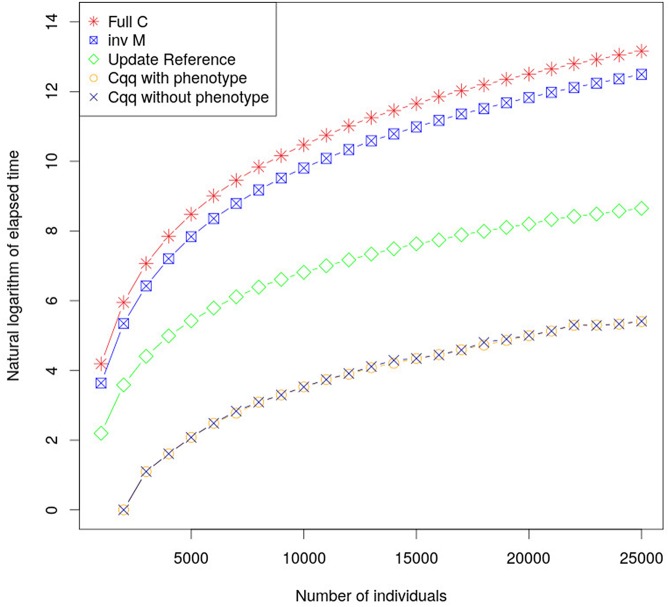
The graph shows the elapsed time required to calculate c^qq^ using different approaches. **inv M** is the elapsed time to calculate the **M** matrix which is the core element to calculate c^qq^ for a new individual. **Update Reference** is the elapse time to update **M** matrix, the **c^qq^** **with and without phenotype** shows the elapsed time to calculate c^qq^ when there is or there is not phenotype for the individual, respectively. Their performances were very similar, and as such the lines overlap.

By calculating the accuracy of young individuals using Equations (14) and (15) computational times have been significantly reduced. Computational performance using this method is considerably faster, in comparison with existing methods, as shown in Figure 1, with only negligible differences in accuracy due to rounding errors (less than 8.88 × 10^−16^). The proposed approach using **Z**′**Z** (a diagonal matrix) resulted in shorter time to build the matrices used to update c^qq^ compared to when using the classic approach to calculate accuracies. This method can be extended in order to accommodate fixed effects and dense **Z′Z** when c^qq^ is updated. Furthermore, the part of **C** for animals with phenotype (**C**^pp^) must be updated as more individuals are phenotyped.

This method could be exploited within routine breeding value estimation for expidited accuracy calculations. Breeding value accuracy is based on an individual's relatedness to the core reference, such that high accuracy indicates high relatedness. This method to calculate accuracy will affect how the genotypes are used, based on how informative they are for the prediction, improving efficiency by reducing redundant information.

New individuals with phenotypes and low accuracy can be added to the core population, as it is likely these animals are lowly related. Their addition improves the diversity and informativity of the core reference population, and can further improve imputation accuracy of the missing genotypes, with added diversity into the imputation haplotype library. Individuals with high accuracy are not required to be added to the core, with or without phenotype, as their accuracy indicates their relatives are already included in this reference population, making their addition redundant. New individuals without phenotypes and low accuracy, should have relatives genotyped to improve accuracy and/or should have their phenotypes recorded to improve the core population.

It is possible to exploit the accuracy calculation as a type of quality control filter for population data, such that individuals with an expected level of relatedness to the reference population, obtains a low accuracy, this may be indicative of genotyping/sampling error, mis-assigned breed, etc. The rapid accuracy calculation for those individuals without phenotype can provide important context for quickly developing a phenotyping strategy.

## 4. Conclusion

Updating the inverse of **C** for new individuals with and without phenotype, using the method here, is shown to reduce the computational effort significantly. With increasing numbers of genotyped animals in genetic evaluations, computational efficiency is essential for frequent and timely evaluations. This method provides an improved and efficient method to deliver accurate and fast evaluations when few new young individuals are genotyped but may or may not have phenotypes.

## Author Contributions

MF developed the method, structured the manuscript, and wrote the method and theory. NC wrote the introduction, result and discussion, and performed major revision. BT gave some comments to improve the final method and article.

### Conflict of Interest Statement

The authors declare that the research was conducted in the absence of any commercial or financial relationships that could be construed as a potential conflict of interest.

## Appendix A

R functions that show the prototype.

 
# Create a partition matrix from 4 matrices
bind  <- function(A, B, C, D)
{
  rbind(cbind(A, B), cbind(C, D))
}
 
# Create a zero matrix
zM  <- function(row, col)
{
  matrix(0, nrow = row, ncol = col)
}
 
# Calculate X'X
xpx  <- function(x)
{
  t(x) %*% x
}
 
# Calculate C inverse by using traditional method
tradMethod  <- function(Zp, Zq, Gpp, Gpq, Gqq, Alpha)
{
  A  <- xpx(Zp)
  D  <- xpx(Zq)
  B  <- zM(nrow(A), ncol(D))
  C  <- t(B)
  solve(bind(A, B, C, D) + Alpha *
  solve(bind(Gpp, Gpq, t(Gpq), Gqq)))
}
 
# Calculate Cqq when there are phenotypes for new
# individuals
cqqPhen  <- function(Zp, Zq, Gpp, Gpq, Gqq, Alpha)
{
  Minv  <- solve(Gpp + Alpha * solve(xpx(Zp)))
  n1  <- -t(Gpq) %*% Minv %*% Gpq + Gqq
  n2  <- (Gqq + Alpha * solve(xpx(Zq)) - t(Gpq) %*%
  Minv %*% Gpq) %*% xpx(Zq)
  n1 %*% solve(n2)
}
 
# Calculate Cqq when there are no phenotypes for
new individuals
cqq  <- function(Zp, Zq, Gpp, Gpq, Gqq, Alpha)
{
  Minv  <- solve(Gpp + Alpha * solve(xpx(Zp)))
  n1  <- -t(Gpq) %*% Minv %*% Gpq + Gqq
  n1 / Alpha
}
 

## References

[B1] Ferdosi M. Connors N. Tier B. (2018). “An efficient method to calculate accuracy of estimated breeding values for individuals without phenotypes,” in Proceedings of the World Congress on Genetics Applied to Livestock Production, Electronic Poster Session - Methods and Tools - Models and Computing Strategies (Auckland).

[B2] Hancock T. P. (2017). “Approximate gblup for efficient routine evaluations,” in Association for the Advancement of Animal Breeding and Genetics (Townsville, QLD).

[B3] Harville D. A. (1997). Matrix Algebra From a Statistician's Perspective, Vol. 1. Yorktown Heights, NY: Springer.

[B4] Henderson C. R. (1963). Selection index and expected genetic advance. Stat. Genet. Plant Breed. 982, 141–163.

[B5] Henderson C. R. (1975). Best linear unbiased estimation and prediction under a selection model. Biometrics 31, 423–447. 1174616

[B6] Henderson H. V. Searle S. R. (1981). On deriving the inverse of a sum of matrices. SIAM Rev. 23, 53–60.

[B7] Meyer K. Tier B. Graser H.-U. (2013). Updating the inverse of the genomic relationship matrix. J. Anim. Sci. 91, 2583–2586. 10.2527/jas.2012-6056 23508030

[B8] Misztal I. (2016). Inexpensive computation of the inverse of the genomic relationship matrix in populations with small effective population size. Genetics 202, 401–409. 10.1534/genetics.115.182089 26584903 PMC4788224

[B9] Sanderson C. Curtin R. (2016). Armadillo: a template-based c++ library for linear algebra. J. Open Source Softw. 1:26 10.21105/joss.00026

[B10] VanRaden P. M. (2008). Efficient methods to compute genomic predictions. J. Dairy Sci. 91, 4414–4423. 10.3168/jds.2007-0980 18946147

